# Nosodes*Read before I. H. A., June 14th, 1884.

**Published:** 1884-09

**Authors:** Edward Bayard

**Affiliations:** New York


### NOSODES.*

EDWARD BAYARD, M. D., NEW YORK.

The law of Homœopathy that like cures like has been invoked to give authority to the practice of administering the product of disease to cure the same disease, and these remedies have been termed nosodes.

There is nothing singular, in fact, of this new word since all the diseases flesh is heir to may enter into each pseudo specific. Drugs have no curative or healing properties in themselves and no direct curative action, whether administered in homœopathic or allopathic doses.

The contrary of this proposition cannot be maintained without establishing that not one but all drugs have directly opposite action in large and small doses, that Arsenic, Lachesis, Belladonna, Nux vomica, and other potent drugs, which, in appreciable quantities are poisons, cease to be such, or in effect have a reverse nature, when used in high potencies or dilutions; and, as a corollary to this proposition, it follows, that between the maximum and minimum doses, between the poisonous and beneficial action of drugs, there must be some neutral point where they cease to have either effect, or at least where their effect passes from the deleterious to the beneficial, that drugs have positive and negative poles, and act upon the body accordingly.

Any attempt to establish these propositions either in theory or practice results in their refutation and rejection.

---

* Read before I. H. A., June 14th, 1884.

On the contrary, the whole homœopathic *materia medica*, the whole of the symptology, is founded upon the inexorable law that the drug in every stage, from the crude to the millionth potency or dilution, possesses the same indestructible and unvarying and unchangeable qualities and action.

Samuel Hahnemann, the profound thinker and earnest searcher after truth, the discoverer of the universal law of Homœopathy, has declared that like cures like, not the same, and in that distinction, well taken, he is sustained by the reason of the thing. Some practitioners of Homœopathy have supposed that they approached nearer the similitude when they adopted the isopathic treatment, giving the virus of smallpox to cure smallpox; the virus of syphilis to cure syphilis, etc. Yet Hahnemann has declared it is not the same that cures.

"The homœopathic system of medicine never pretended to cure a disease by the *same*, the *identical*, agent by which the disease was produced. This has been inculcated on the unintelligent opponents often enough, but, as it seems, in vain. No! it only cures by means of an agent never exactly corresponding to, never *identical* with, the cause of the disease, but by means of a medicine that professes the peculiar power of being able to produce only a *similar* morbid state (ὅμοιον πάθος) and this is the mode most in conformity with nature."

"Cannot these persons feel the difference between 'identical' (the same) and 'similar'?"—Nota bene to Hahnemann's *Materia Medica Pura*, Vol. 2.

The attempt to obtain from the body, specifics for the diseases of the body, is liable to two grave objections.

First. It proceeds upon the erroneous assumption that cure is wrought by identicals, not by similars. *Similia similibus curantur.*

The system always has a tendency to react against the morbific cause which disturbs it. The nosode, the same, leads this reaction into the vortex of the disease.

When we consider that the law of cure is by stimulating reaction through the vital force, it will appear that the temporary reaction which is aroused by the identical must sooner or later be lost in the greater force already exerted by the disease; their actions being identical, they must unite and the greater absorb the less, whereas the similar raises a parallel reaction which never becomes identical with or merged in the action of the disease, but passing beyond it, ends in the equalization of the vital force, wherein the reaction equals the action, which is the state of perfect repose and perfect health. The tendency is to react against the disease; if those forces directly opposed to the disease are overwhelmed they cannot unaided react; it is only by calling in aid the correlated yet unaffected powers of resistance that you effect an alliance capable of successful reaction; to this end you administer a similar; the nearer the relations of the similar to the disease, the closer and more united will be the powers of reaction, the more speedy the cure.

This power of reaction depends not upon the strength of the dose but upon the exactitude of the prescription. The larger the dose the more deleterious its direct action. The smallest dose compatible with the stimulus to reaction is the best. The higher the potency the more speedy the reaction. It is in vain, then, to seek to extract from a diseased part a pure and homogeneous remedy which shall cure the same disease in others.

Second. Even if it could be done theoretically, practically, the nosode would contain the seeds of hereditary evils, which had no visible exponent in the disease expressed, and while antidoting one disease you might inoculate with the virus of many others. To avoid this danger, the cowpox was substituted for the smallpox. There are fewer hereditary diseases among animals, and of these few are common to humanity. By the use of these nosodes Homœopathy would justly incur the reproach of "monstrous polypharmacy," which Sir John Forbes hurled at the allopathic school, of which he was one of the heads.

It is true that the nosode does make a new impression and arouses a temporary reaction, but it is soon overborne by the more powerful action of the disease. From its nature it cannot complete a cure, it cannot destroy what it has created. The gain is illusory, and the danger of transplanting the seeds of hidden hereditary evils should be a sufficient deterrent for every true physician.

In his inaugural address, delivered before the Health Congress of Brighton, England, Dr. W. B. Richardson said, speaking of hereditary diseases: "I am satisfied that quinsy, diphtheria, scarlet fever, and even what is called drain fever, typhoid, are often of hereditary character. I have known a family in which four members have suffered from diphtheria, a parent having had the same affection, and probably grandparent. I have known a family of which five members have, at various periods, suffered from typhoid, a parent and grandparent having been subject to the same disease. I have known a family in which quinsy has been the marked characteristic for four generations. These persons have been sufferers from the diseases named without any obvious contraction of the diseases and without having any companions in their sufferings. They were, in fact, predisposed to produce the poisons of the disease in their own bodies, as the cobra is to produce the poisonous secretion which in this case is a part of its natural organization."

